# Enhancing photoluminescence of carbon quantum dots doped PVA films with randomly dispersed silica microspheres

**DOI:** 10.1038/s41598-020-62563-1

**Published:** 2020-03-31

**Authors:** Xun Zhao, Ailin Wang, Sili Gao, Duanting Yan, Wanying Guo, Yingyue Xu, Yanli Meng, Chunliang Wang, Guiye Shan

**Affiliations:** 1 Center for Advanced optoelectronic Functional Materials Research and Key Laboratory for UV light-Emitting Materials and Technology of Ministry of Education, Northeast Normal University, Changchun, 130024 PR China; 2 Key Laboratory of Infrared System Detection and Imaging Technology, Shanghai Institute of Technical Physics, Chinese Academy of Sciences, Shanghai, 200083 PR China

**Keywords:** Nanoparticles, Quantum dots, Polymers, Micro-optics

## Abstract

As a kind of excellent photoluminescent material, carbon quantum dots have been extensively studied in many fields, including biomedical applications and optoelectronic devices. They have been dispersed in polymer matrices to form luminescent films which can be used in LEDs, displays, sensors, etc. Owing to the total internal reflection at the flat polymer/air interfaces, a significant portion of the emitted light are trapped and dissipated. In this paper, we fabricate free standing flexible PVA films with photoluminescent carbon quantum dots embedded in them. We disperse silica microspheres at the film surfaces to couple out the total internal reflection. The effects of sphere densities and diameters on the enhancement of photoluminescence are experimentally investigated with a homemade microscope. The enhancement of fluorescence intensity is as high as 1.83 when the film is fully covered by spheres of 0.86 ${\boldsymbol{\mu }}$m diameter. It is worth noting that the light extraction originates from rather the scattering of individual spheres than the diffraction of ordered arrays. The mechanism of scattering is confirmed by numerical simulations. The simulated results show that the evanescent wave at the flat PVA/air interface can be effectively scattered out of the film.

## Introduction

Carbon quantum dots (CDs) have attracted significant attention in recent years^1,2^. They have been widely applied in fields such as light emitting diodes(LEDs)^3,4^, solar cells^5^, photocatalysts^6,7^, electrochemical sensing^8^, bioimaging^9^, photodetectors^10^, lasing^11^, and photothermal therapies^12^. They can be synthesized with facile and low cost methods such as electrochemical fabrication^7^ and solution phase synthesis^5^. They can also be made from low cost raw materials, including egg yolk oil^13^, waste papers^14^ and tomatoes^15^. CDs have merits of long term stability^16^, low environmental and biological toxicity^9^, broad band optical absorption^17^ and good electrochemiluminescence activity^18^. As a kind of promising photocatalyst, CDs have shown excellent performances in photoelectrochemical and photocatalytic applications due to their semiconductor-like photoelectric properties^6^. CDs can be combined with other materials to obtain nanocomposites with excellent optoelectronic properties. Surface plasmon resonance has been demonstrated in carbon-dot-supported silver nanoparticles^19^. Such composites benefit from excellent electron-donating capability of photoexcited carbon dots. The resulted clustering effect of silver particles can modify the plasmonic properties and lead to dramatic efficiency enhancement of optelectronic devices. A ruthenium nanoparticles@CDs hybrid material exhibited remarkable catalytic ability for hydrogen evolution reaction with good stability and catalytically durability^20^.

One of the most attractive properties of CDs is their strong and tunable photoluminescence^21,22^(PL). The formation mechanism and fluorophores have been extensively discussed^2^, and the wavelengths of emission can be tuned by components and synthesis procedures. In CDs synthesized through controlled thermal pyrolysis of citric acid and urea, the maximum emission was gradually tuned from blue to red by regulating the thermal-pyrolysis temperature and ratio of reactants^23^. CDs synthesized by a one-step hydrothermal process have been shown to exhibit broad band emission over the whole visible wavelength range^24^. All the emission centers were excited by a single ultraviolet source due to aggregations and Förster resonance energy transfer between the CDs. Excitation-independent near-infrared emission has also been demonstrated owing to surface chemical states and homogeneous microstructures of the CDs^25^. The PL properties of CDs can be further improved with physical mechanisms. For instance, the green and yellow emission of CDs was enhanced by localized surface plasmon resonance of Ag nanoparticles^26^.

In order to explore their applications in optoelectronic devices, it is favorable to make CDs in solid forms^27^. A simple way is to disperse CDs in polymer matrices which can protect them from concentration-induced PL self-quenching. It was demonstrated that CDs could be well dispersed into epoxy matrices and the CDs/epoxy composites could be applied to encapsulate LED chips^23^. Flexible full-color emissive poly vinyl alcohol (PVA) films have been achieved through mixing two or three CDs in appropriate ratios^28^. Moreover, incorporating CDs in polymer matrices can also improve their luminescent properties. Comparing to the aqueous solutions, CDs dispersed in PVA films exhibited enhanced fluorescence emissions^29^. The enhancement was attributed to enhanced surface passivation for the carbon dots in a more confined environment in the PVA matrix. CDs are also compatible with other luminescent materials in polymer matrices. When CDs and EuCl${}_{3}$ were dispersed together in a PVA film, the PL spectrum could be tuned by adjusting the mixing proportion of CDs and Eu${}^{3+}$^30^. Based on the polymer films doped with CDs, displays and LEDs with excellent performance can be expected. Besides, the application of CDs doped luminescent polymer films can be further extended to the research of sensors. Stretching of CDs doped polymer films induced both blue shift in the fluorescence peak positions and dramatic increase in fluorescence intensities^31^. Such phenomena can facilitate optical determination of tensile properties.

**Figure 1:**
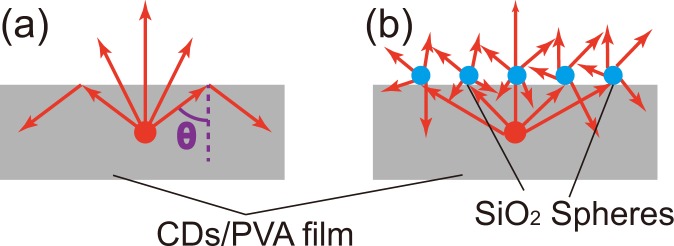
Illustration of light propagation from a luminescent center inside a polymer film, (**a**) bare flat surface, (**b**) with silica microspheres on the surface.

However, there is a drawback of embedding CDs in transparent polymer films. Because the refractive indices of polymers are higher than air, a considerable portion of luminescence from CDs are reflected back by the total internal reflection (TIR) at the polymer/air interface [Fig. 1(a)]. Such a phenomenon can be analyzed with a simple ray-optics model^32,33^. For a polymer with the refractive index of 1.5, the critical angle of TIR is $41.{8}^{\circ }$. Assuming a luminescent center inside the polymer film emits light identically to all the directions, about 74.5% of the light emission toward the PVA/air interface is reflected back by TIR. The reflected light often dissipates through waveguide modes when the bottom of the film is also reflective.

Trapping of light in layers of high refractive indices is a long existed problem in the field of LED^3,33,34^. Many efforts have been devoted to suppression of TIR and extraction of trapped light. The key issue is to eliminate sudden changes of refractive indices at flat interfaces. The efficiency of an organic light-emitting diode (OLED) can be increased by simply roughening the substrate surface by sand blasting^35^. Micro lens arrays were attached to the glass substrate of an OLED, and led to an extraction increase of 60%^36^. Organic particles can serve as scattering media and extract waveguide light^37^. Light extraction can be enhanced by combining the ideas of refractive index matching and photon recycling in films with quantum dots dispersed in them^38^. Irregular subwavelength nanopillars were made on flexible polycarbonate substrates with the nanoimprint method^39^. The efficiency of the OLED was improved by 69% with such an antireflective structure.

Similar to the study of light extraction of LED, structures on surfaces of CDs doped polymer films have also been elaborately designed to enhance the PL efficiency. Using a surface micro-textured silicon wafer as a template, free standing CDs/PVA films with large-area ordered inverted-pyramid patterns were fabricated^40^. Similar to the anti-reflective (AR) surfaces used in solar cells, such a structure reduced the reflection from the PVA/air interface and led to a quantum yield enhancement. Periodic micro-structures were also patterned onto the film surface with a commercial digital versatile disc serving as a mold^41^. The submicron-patterns with a periodicity of 700 nm provided an emission enhancement factor of 1.96. The enhancement was interpreted as the compensation for momentum mismatch between the waveguide-mode light and far-field radiation.

In this paper, we demonstrate the light extraction of CDs/PVA films with silica microspheres [Fig. 1(b)]. The spheres are dispersed on the film surfaces and scatter out the TIR. The scattering is induced by rather individual spheres than ordered arrays, therefore, there is no requirement of periodicity. Because random structures can extract light propagating along any direction with a wide spectral range^42^, the strategy presented here is suitable for broad-band emitting devices.

## Experiments

**Figure 2:**
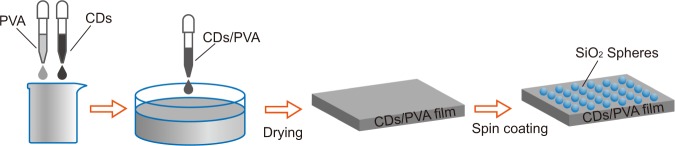
Procedure of preparing CDs/PVA film with silica spheres at the surface.

The fabrication of samples started with preparing CDs and PVA solutions. The CDs were synthesized with a hydrothermal method^43,44^. Urea (0.12 g) and p-phenylenediamine (0.12 g) were dissolved in ultrapure water (30 mL). The solution was heated in a sealed autoclave at 160 ${}^{\circ }$C for 10 hours. After cooling down to room temperature, the obtained solution was filtered with a microporous membrane and dialyzed against water. As for the PVA solutions, 1.5 g of purchased PVA powder [Mw 85,000–124,000, Sigma-Aldrich] was added to ultrapure water (30 mL). The mixture was stirred for about 5 hours until the powder was completely dissolved. The following procedure of making a film was shown in Fig. 2. The solutions of CDs (concentration: 1.5 mg/mL) and PVA (mass concentration: 5%) were mixed at the ratio of 1:100 by volume, then stirred for 5 minutes until a uniform liquid was obtained. The resulting CDs/PVA solution was transferred to a petri dish and left for 15 hours, so that the air bubbles were completely removed. After being dried for 3 days at the temperature of 60 ${}^{\circ }$C, the solution became a uniform solid film. The thickness of the CDs/PVA film was determined by the volume transferred into the petri dish. Finally, the film was cooled down to room temperature then peeled off to be free standing.

Purchased silica microspheres [BaseLine Chromtech Research Centre] with different diameters (0.3 $\mu$m, 0.86 $\mu$m, 1 $\mu$m, and 1.7 $\mu$m) were dispersed on the surfaces of CDs/PVA films by spin coating. The specific process is as follows: firstly, suspensions of microspheres were diluted to be 12.5 mg/mL; secondly, the ultrasonic dispersion method was applied for 10 minutes to make sure the microspheres were monodisperse; finally, the suspensions were spin coated [[Laurell Technologies, WS-650-23]] on top of the films at 400 rpm for 1 minute. In the spin coating step, the PVA films were adhered to glass slides with the help of Polydimethylsiloxane (PDMS) to keep the surfaces flat.

The basic properties of the CDs/PVA films were characterized by commercial instruments. The morphologies of the dispersed microspheres were examined by a scanning electron microscope (SEM) [FEI, Quanta FEG 250]. The absorption was measured with a UV-Vis spectrometer [Macylab instruments, UV-1900], while the PL and excitation spectra were obtained by a spectrofluorophotometer [Shimadzu, RF-6000].

**Figure 3:**
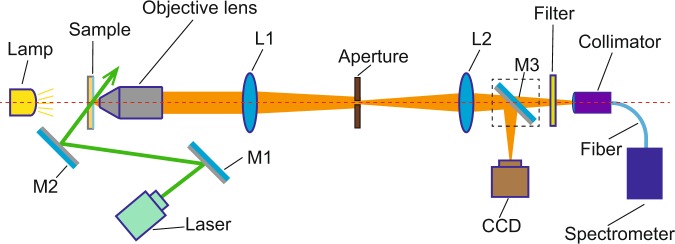
Scheme of the optical setup for the PL measurement.

In order to investigate the PL enhancements of spheres, we built a home made microscope equipped with a high sensitivity fluorescence spectrometer [Ocean Optics, QE Pro]. The configuration of the optical setup was shown in Fig. 3. The illuminator of the sample (CDs/PVA films) can be switched between a halogen lamp and a continuous wave laser. As for the detector, a switchable mirror (M3) was used to choose between a CCD camera and the spectrometer. First, the sample was finely tuned to be at the focus of the objective lens. Then a lens (L1, focal length: 200 mm) projected the image of the sample to its focal plane, where the magnified image was filtered by an adjustable aperture. Finally, the light went through the aperture was refracted by another lens (L2, focal length: 100 mm) and captured by the CCD camera or the collimator followed by the spectrometer. When the sample was illuminated by the lamp and the mirror M3 was in the optical path, the CCD camera detected the image of the film surface. When the sample is illuminated by the laser and the mirror M3 was out of the optical path, the PL spectrum was measured by the spectrometer. The direction of the laser beam (wavelength: 532 nm; power: 200 mW; diameter: 1 mm at the position of the sample) was tuned with two mirrors M1 and M2. The CDs was excited by the green laser and emitted yellow fluorescence. A Long-Wavelength-Pass filter was used to block the scattered laser. PL from the part of the film selected by the aperture was collected by the collimator then analyzed by the spectrometer.

## Numerical Simulations

**Figure 4:**
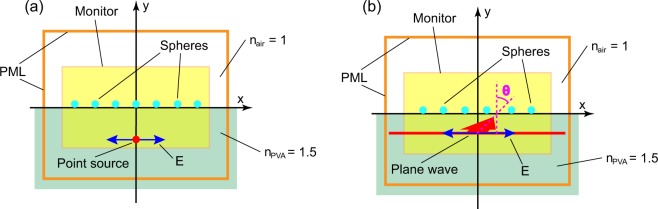
Model used for numerical simulations, the light sources are (**a**) a point source, (**b**) a plane wave.

Since the diameters of the microspheres were in the $\mu$m range, we resorted to the electromagnetic wave theory to analyze the scattering mechanism. A 2-D finite-difference time-domain (FDTD) method [Lumerical FDTD] was conducted to simulate the behavior of electromagnetic waves. The theoretical models used in the numerical calculations are shown in Fig. 4. In the $x-y$ plane, the refractive indices in the areas of $y\le 0\ \mu$m and $y > 0\ \mu$m were set to be the refractive indices of PVA and air respectively^45^. The size of simulation area surrounded by perfectly matching layers (PML) was set to be $100\ \mu \,{\rm{m}}\ast 20\ \mu$m ($-50\ \mu \,{\rm{m}}\,\le x\le 50\ \mu$m, $-10\ \mu \,{\rm{m}}\,\le y\le 10\ \mu$m). The distribution of electric field density was recorded by a frequency-domain field monitor. We simulated two different cases with the light sources being a point source [Fig. 4(a)] and a plane wave source [Fig. 4(b)], respectively. In the first case, the point source was placed 5 $\mu$m below the PVA/air interface. The size of the monitor was set to be $20\ \mu \,{\rm{m}}\ast 10\ \mu$m ($-10\ \mu \,{\rm{m}}\,\le x\le 10\ \mu$m, $-5\ \mu \,{\rm{m}}\,\le y\le 5\ \mu$m). In the second case, a plane wave source with the span of $100\ \mu$m ($-50\ \mu \,{\rm{m}}\,\le x\le 50\ \mu$m) was placed $2\ \mu$m below the PVA/air interface. The propagation direction was set to be $\theta =4{5}^{\circ }$to the normal of the interface. TM modes were used in all the simulations.

## Results and Discussions

**Figure 5:**
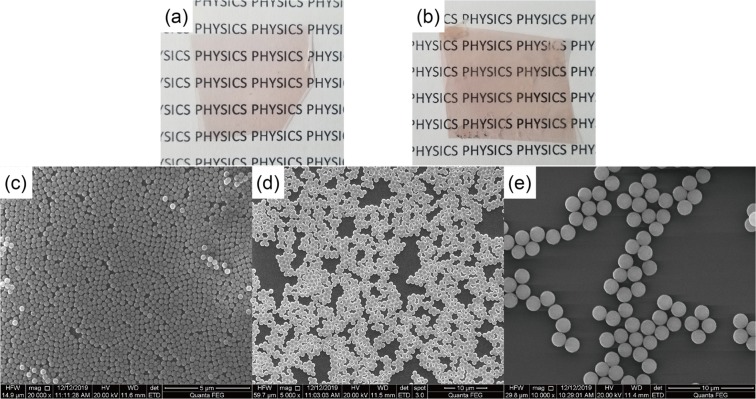
Photos and SEM pictures of prepared PVA films. (**a**) film without microspheres; (**b**) film with silica microspheres of 0.86 $\mu$m diameter; (**c**) SEM picture of close packed microspheres, the sphere diameter is 0.3 $\mu$m; (**d**) SEM picture of microspheres aggregating at different places, the sphere diameter is 0.86 $\mu$m; (**e**) SEM picture of spheres aggregating in small groups, the sphere diameter is 1.7 $\mu$m.

Transparent and flexible films were fabricated following the aforementioned process [Fig. 2]. All the films have smooth flat surfaces before spin coating microspheres. Two typical CDs/PVA samples without and with microspheres (diameter of 0.86 $\mu$m) are shown in Fig. 5(a,b). Owing to the scattering, the transmittance of light decreases for the film with microspheres [Fig. 5(b)].

For the investigation of scattering induced by dispersed microspheres, the parameters of the spin coating process were controlled to make monolayers of the microspheres. Due to the fact that the dispersions of microspheres are not perfectly uniform, we can find different dispersions at different parts of a film. Figure 5(c–e) display three representative situations of the obtained microspheres. In Fig. 5(c), the observed surface is completely covered by microspheres. The spheres in most parts are in the close packed form with occasional vacancies among them. In Fig. 5(d), a large fraction of the area is covered by microspheres. All the spheres are in a mono layer, and form aggregations. Each aggregation comprises of tens of spheres, and periodicity can hardly be found. In Fig. 5(e), only a small fraction of the area is covered by microspheres. Several spheres aggregate in small groups along random directions. We can find these cases in all the films with microspheres of different diameters. That enables us to select different areas to investigate the effects of different microsphere dispersions.

**Figure 6:**
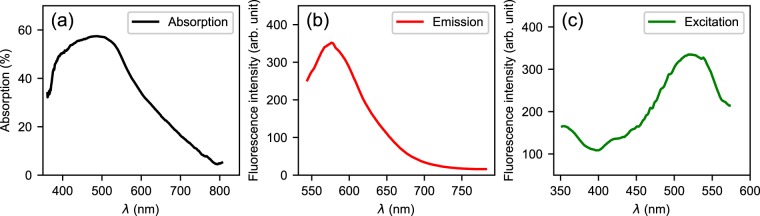
Optical properties of the CDs/PVA films. (**a**) absorption spectrum, (**b**) fluorescence spectrum at the excitation wavelength of 532 nm, (**c**) Excitation spectrum at the emission wavelength of 585 nm.

The basic optical properties of the prepared CDs/PVA films are shown in Fig. 6. Because 532 nm is one of the most common used laser wavelengths, we pay special attention to the absorption and excitation at this wavelength. The CDs in the PVA matrix exhibit broad band absorption over the visible wavelength range [Fig. 6(a)]. The absorption peak lies at about 489 nm, while the absorption at the wavelength of 532 nm is still high. At the excitation wavelength of 532 nm, the films emit yellow fluorescence shown in Fig. 6(b). The emission has a broad spectral profile spanning over 100 nm. Monitoring the emission intensity at 585 nm, the measured excitation spectrum is shown in Fig. 6(c). The PL can be induced by a wide range of wavelengths, especially, the excitation efficiency of 532 nm is near the peak value.

**Figure 7:**
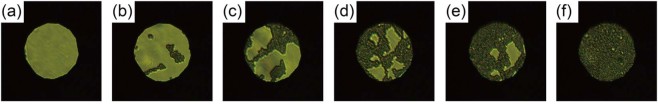
CDs/PVA film covered by spheres. The spheres densities are (**a**) 0, (**b**) 20%, (**c**) 40%, (**d**) 60%, (**e**) 80%, (**f**) 100%.

Although the resolution of an optical microscope is not high enough to see the details of a microsphere, the dispersions of spheres can be seen by our home made setup [Fig. 3]. Using a $40\times$ objective (NA = 0.65), a circular field with 0.25 mm diameter on the film surface was imaged. At the focal plane of L1, the diameter of the image was about 10 mm. In order to precisely observe a small area, the diameter of the adjustable aperture was tuned to be 2 mm. Therefore, we can select a small circular field with 50 $\mu$m diameter. By transversely moving the sample, we can select different parts of the film surface and find different sphere densities [Fig. 7]. Before measuring the PL spectrum, we used an open source software^46^ [ImageJ] to measure the area of covered/bare parts (see Supplementary Fig. S1 online), then carefully tuned the sample position until we got expected sphere densities. When the selected area is completely covered by the spheres [Fig. 7(e)], the sphere density is defined as 100%. We use different sphere densities (20%, 40%, 60%, 80%, 100%) to quantitatively investigate the PL enhancements induced by microspheres. During measurements of each film, the spectrum of a selected bare area without microspheres [Fig. 7(a)] was used as a reference. In this way, differences between different CDs/PVA films were avoided.

**Figure 8:**
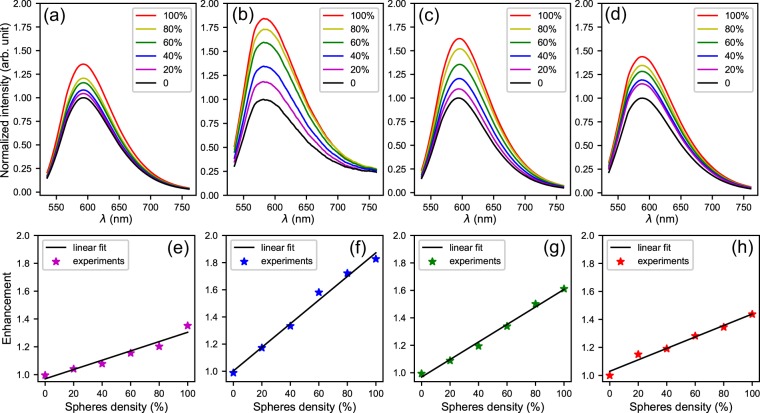
Normalized PL spectra and enhancement factors corresponding to different sphere densities. The diameters of the microspheres are (**a,e**) 0.3 $\mu$m, (**b,f**) 0.86 $\mu$m, (**c,g**) 1 $\mu$m, (**d,h**) 1.7 $\mu$m.

In the measurements of PL spectra, the laser beam entered the CDs/PVA films from the back side (the surface without microspheres) and was not affected by microspheres, so that the CDs were always excited identically. The incident angle of the laser was carefully adjusted to be about $6{0}^{\circ }$ to avoid multi reflections inside the films (in the measured area). The measured PL spectra of CDs/PVA films with different sphere densities and diameters are shown in Fig. 8. Figure 8(a–d) are the spectra corresponding to sphere diameters of 0.3 $\mu$m, 0.86 $\mu$m, 1 $\mu$m, and 1.7 $\mu$m, respectively. In each sub figure, the fluorescence intensities are normalized to the maximum of the bare surface [black lines]. The purple, blue, green, olive, and red lines are normalized PL spectra corresponding to sphere densities of 20%, 40%, 60%, 80%, and 100%, respectively.

For the microspheres of each diameter, PL spectra are almost the same for different sphere densities. That means the microspheres scatter light almost identically over a broad range of wavelengths. This phenomenon is in accordance with the fact that there is no large ordered arrays on the surfaces [Figs. 5 and 7]. Ordered arrays of dielectric microspheres tend to form photonic crystals whose diffractions are sensitive to wavelengths and directions^47^. By contrast, randomly dispersed microspheres are suitable for the applications requiring broad band performance.

Light extraction induced by the microspheres are prominent. Comparing to the bare film surfaces (black lines), the intensities increase dramatically with increasing sphere densities. The maximum of PL enhancements appears at the diameter of 0.86 $\mu$m for 100% sphere density. The normalized intensity at the wavelength of 585 nm is as high as 1.83.

The light extraction is attributed to the scattering of individual microspheres. This is confirmed by the dependence of PL enhancements on sphere densities. For each sphere diameter, we extract the maximums of the normalized intensities for different sphere densities, and plot them in Fig. 8(e–h). The relationships between these values and sphere densities can be well linearly fitted (black lines) with the slopes being 0.33, 0.87, 0.64, and 0.41, respectively. Considering microspheres aggregate in different ways at different sphere densities, there is little influence of aggregation on the light extraction.

When the sphere density is 100%, which means the surface is completely covered with spheres, the maximum of normalized PL intensities are 1.35, 1.83, 1.61, and 1.44 corresponding to the diameters of 0.3 $\mu$m, 0.86 $\mu$m, 1 $\mu$m, and 1.7 $\mu$m, respectively. From both the slopes and the maximums of normalized intensities, it is clear that the enhancement varies with different sphere diameters. The microspheres with the diameter of 0.86 $\mu$m show the best enhancing effects. The smaller and bigger spheres are less effective in light extraction.

**Figure 9:**
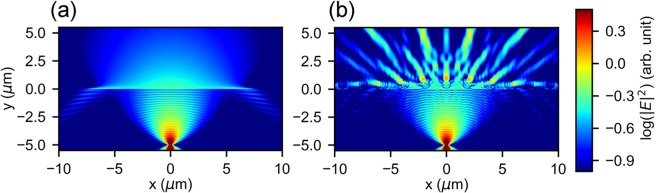
Calculated distribution of electric field intensity near PVA/air interface under the illumination of a point source. (**a**) bare interface, (**b**) with micropheres attached to the interface.

**Figure 10:**
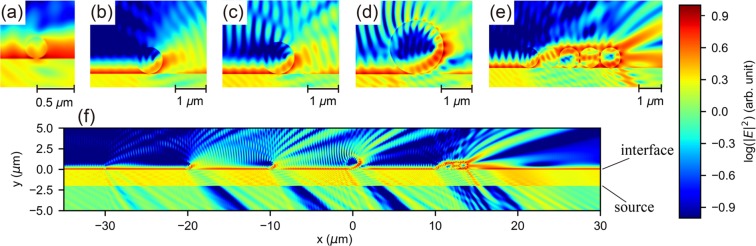
Calculated distribution of electric field intensity near PVA/air interface under the illumination of a plane wave source. (**a–d**) Intensity distribution near single spheres, the diameters are (**a**) 0.3 $\mu$m, (**b**) 0.86 $\mu$m, (**c**) 1 $\mu$m, (**d**) 1.7 $\mu$m. (**e**) Intensity distribution near a 5-spheres aggregation, the diameter of the spheres is 0.86 $\mu$m. (**f**) Intensity distribution near the PVA/air interface in a wide scope ranging from $x=-30\ \mu$m to $x=30\ \mu$m.

The observed PL enhancements mainly come from destroying the TIR. The electric field distribution near the spheres show the effects of spheres on the light propagation [Figs. 9 and 10]. Assume a point source located at the position of $x=0\ \mu \,{\rm{m}}\,,y=-5\ \mu$m, from where the electromagnetic wave propagates toward the PVA/air interface ($y=0\ \mu$m). In the case of the bare film [Fig. 9(a)], the electric field intensity (denoted with different color corresponding to $\,{\rm{\log }}\,| E{| }^{2}$) decays smoothly with the propagation distance. There is an obvious contrast of the field intensity below and above the interface because of the change of refractive index. When 11 microspheres are placed at the interface, the distribution of field intensity is modified dramatically by the spheres [Fig. 9(b)]. In order to show the contribution of individual spheres, the distance between adjacent spheres was set to be 1 $\mu$m. The diameter of the spheres was set to be 0.86 $\mu$m. It is clear that each sphere induces significant scattering of the electromagnetic wave. The main mechanism of PL enhancement is the extraction of reflected light at large incident angles. When looking into the areas of $x < -5\ \mu$m and $x > 5\ \mu$m below the interface, we can see that the intensity reflected downward [Fig. 9(a)] decrease with the addition of spheres [Fig. 9(b)].

The effects of silica microspheres on TIR are further shown in Fig. 10. The wavelength of the plane wave source was set to be 585 nm [Fig. 4(b)]. At the incident angle of 45${}^{\circ }$, TIR happens at the flat PVA/air interface. In order to investigate the effects of individual spheres on the electric field distribution, four spheres with diameters of 0.3 $\mu$m, 0.86 $\mu$m, 1 $\mu$m, and 1.7 $\mu$m were placed at the PVA/air interface. An aggregation of 5 spheres with the diameter of 0.86 $\mu$m was also placed at the interface to show the collective effects of sphere. The positions of these spheres were set to be $x=-30\ \mu$m, $x=-20\ \mu$m, $x=-10\ \mu$m, $x=0\ \mu$m, and $x=10\ \mu$m. In such a configuration, the spheres do not influence each other because the distances between them are far larger than the wavelength. Figure 10(a–e) are the calculated electric field distribution near these spheres.

When TIR happens, there are evanescent waves^48^ penetrating into air with a limited spatial distance (in the order of wavelengths). The scattering is induced by the following processes: the evanescent wave is coupled into silica microspheres, then re-emitted out of the spheres. The profiles of evanescent wave can be seen from the red parts near the interface in Fig. 10. The sphere with the diameter of 0.3 $\mu$m is so small, that it induces small modifications of the evanescent wave [Fig. 10(a)]. When the diameters become larger, the spheres lead to significant modifications of the evanescent wave [Fig. 10(b–d)]. As the plane wave propagates from left to right, the evanescent wave is effectively coupled into the spheres to the right of the contact points. If several microspheres aggregate together, the profile of the evanescent wave is different from the case of single spheres [Fig. 10(e)]. A great part of the evanescent wave near the aggregation is still coupled into the spheres and re-emitted into air.

The electric field distribution in a wide scope is shown in Fig. 10(f). In the places far from the microspheres, there is a uniform evanescent wave near the interface. The evanescent wave near the microspheres is significantly scattered. Subsequently, we can see the pattern of scattered intensities in the region of air ($y > 0\ \mu$m). This is the main origin of light extraction. The effects of microspheres can also be seen in the region of PVA ($y < 0\ \mu$m). Because the plane wave source is placed at $y=-2\ \mu$m, the electric field in the region of $-2\ \mu \,{\rm{m}}\, < y < 0\ \mu$m is determined by the superposition of incident and reflected waves, while the electric field in the region of $y < -2\ \mu$m is only induced by the reflected wave. In these two regions, we can see clearly intensity reductions originating from the microspheres, because the reflection wave is scattered.

The sphere diameter is an important parameter of scattering^49^. Scatterings increase with increasing diameters, therefore, PL enhancements induced by microspheres of 0.86 $\mu$m diameter are higher than 0.3 $\mu$m diameter. However, the evanescent wave decays rapidly as the distance to the interface increases. It can only be coupled into the spheres when silica is near the interface. If a sphere is too big, most part of the sphere becomes too far from the interface, and less evanescent wave is coupled into it. This is the reason for decreased PL enhancements of bigger spheres (1 $\mu$m and 1.7 $\mu$m diameters).

From the experimental and simulated results, we can see that the most important parameter of PL enhancement is the density of microspheres. In order to get the best performance of PL enhancement, the polymer surface should be covered with microspheres as much as possible. Sphere diameter is another important factor. PL enhancement can be optimized by choosing proper sphere diameters.

Silica microspheres have been used as AR layers on top of transparent substrates^47^, but the mechanism was different from the light extraction investigated in this paper. When the surface of a substrate was covered by hexagonally closely packed microspheres, the spheres layer was viewed as a thin film comprising of silica spheres and air. The effective refractive index (1.30) was calculated from the dielectric constant and filling factor of silica. The spheres layer reduced the refractive index contrast between the substrate and air, and led to the suppression of Fresnel reflection. However, the AR effect worked only at small incident angles, TIR could not be affected by the AR layers. Besides, the desired diameter of the spheres, which defined the thickness of the AR layer, was a quarter of the wavelength. In this case, the reflection at the substrate/spheres and spheres/air interfaces interfere destructively at normal incidence. Incorporating a thin film with the refractive index being 1.30, we calculated the reflection of a PVA/film/air structure with a transfer matrix method^50^ (see Supplementary Fig. S2 online). At the wavelength of 585 nm and incident angles smaller than the critical angle (about 42${}^{\circ }$) of TIR, the thin film showed the best AR effect at the thickness of 113 nm. When the incident angles were larger than the critical angle, the reflection was 100% at all the film thicknesses (113 nm, 300 nm, 860 nm, 1000 nm, and 1700 nm).

Hexagonally closely packed microspheres were used to extract trapped light in OLEDs^51^. The microsphere arrays acted as a two dimensional diffraction lattice, which effectively scattered out the wave guided modes. It was demonstrated that the scattering was highly dependent on both the wavelength and diffraction angle. The emission spectra were significantly modified by the periodic structures. Rainbow patterns at the emission zones were also observed. The microspheres in this paper are randomly dispersed, and there is no long-range periodic structure. As a result, the effects of wavelength and direction on PL enhancement are negligible. Since the PL enhancements do not depend on periodicity, the microspheres can be useful in wearable and stretchable devices^52,53^. Comparing to the ordered structures^36,47^, randomly dispersed spheres are much easier to fabricate. Neither complex processes (such as etching) nor rigorous conditions (such as vacuum) are needed. In order to fabricate polymer films with structured surfaces, the fabrication processes are often specially designed from the beginning^41^, while the microspheres can be dispersed on fabricated film surfaces. Therefore the strategy demonstrated here can be included in established technologies. Additionally, functions such as self cleaning^54^ can also be explored based on dispersed silica microspheres. The dispersion of dielectric microspheres can also be used in other luminescent films such as inorganic semiconductor doped polymers^55^, dye doped polymers^56^, and OLEDs.

## Conclusion

We have dispersed CDs in the matrices of free standing PVA films, and fabricated randomly dispersed silica microspheres on the surfaces. The scatterings of microspheres lead to significant PL enhancements (maximum is 1.83). With an aperture superimposed on the intermediate images of film surfaces, the fluorescence spectra have been investigated in a small selected area (25 $\mu$m diameter). The experimental results show that the PL enhancements depend linearly on the sphere densities. The PL enhancements induced by the spheres of 0.86 $\mu$m diameter is higher than smaller (0.3 $\mu$m) and bigger (1 $\mu$m and 1.7 $\mu$m) spheres. The enhancements are attributed to the extraction of TIR by scatterings of individual silica microspheres. The effects of microspheres on the near-field distribution of electric field are analyzed with the FDTD method. The strategy demonstrated here is easy to implement because no ordered pattern is required.

## Supplementary information

Supplementary Information.
