# Supplementary material for: Enhancing photoluminescence of carbon quantum dots doped PVA films with randomly dispersed silica microspheres
Source: Sci Rep. 2020 Mar 31;10:5710. doi: 10.1038/s41598-020-62563-1 (PMC7109140; doi:10.1038/s41598-020-62563-1)
Supplement: Supplementary file 1 — Supplementary Information. [file 41598_2020_62563_MOESM1_ESM.pdf]

Enhancing photoluminescence of carbon quantum dots doped PVA films with randomly dispersed silica microspheres

Xun Zhao[1], Ailin Wang[1], Sili Gao[2], Duanting Yan[1], Wanying Guo[1], Yingyue Xu[1], Yanli Meng[1], Chunliang Wang[1*], and Guiye Shan[1*]

[1]Center for Advanced optoelectronic Functional Materials Research and Key Laboratory for UV light-Emitting Materials and Technology of Ministry of Education, Northeast Normal University, Changchun 130024, PR China.

[2]Key Laboratory of Infrared System Detection and Imaging Technology, Shanghai Institute of Technical Physics, Chinese Academy of Sciences

*wangcl493@nenu.edu.cn; *shangy229@nenu.edu.cn

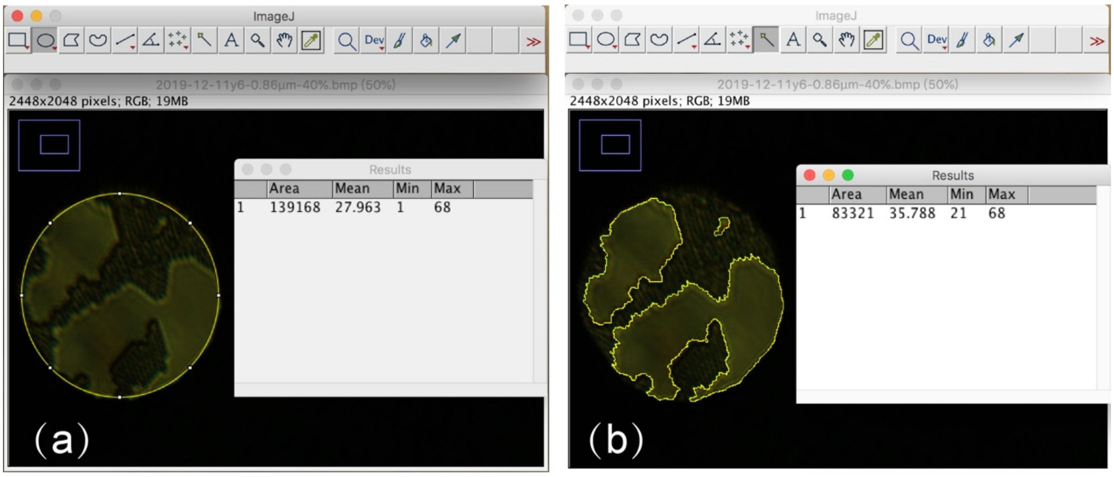

Supplementary Figure S1: Measurement of areas with the software ImageJ. (a) the whole circular area, (b) parts without microspheres.

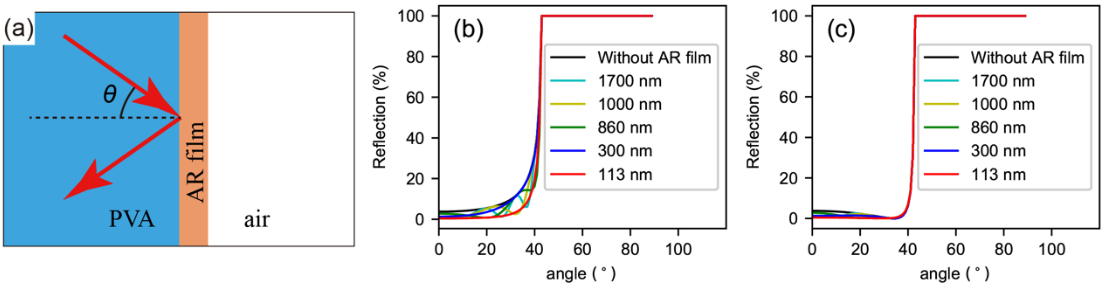

Supplementary Figure S2: Calculated reflection of a PVA/film/air structure. (a) model used for calculation, (b) reflection of TE mode, (c) reflection of TM mode.
